# A patient-specific proof of concept with a three-dimensionally printed model before performing an endovascular Bentall procedure

**DOI:** 10.1016/j.jvscit.2021.09.014

**Published:** 2021-10-14

**Authors:** Aurelien Vallée, Julien Guihaire, Said Ghostine, Dominque Fabre, Stephan Haulon

**Affiliations:** a Aortic Center, Marie Lannelongue Hospital, Groupe Hospitalier Paris Saint-Joseph, Le Plessis Robinson, France; b Vascular Surgery Department, Marie Lannelongue Hospital, Groupe Hospitalier Paris Saint-Joseph, Le Plessis Robinson, France; c Cardiac Surgery Department, Marie Lannelongue Hospital, Groupe Hospitalier Paris Saint-Joseph, Le Plessis Robinson, France; d Interventional Cardiology Department, Marie Lannelongue Hospital, Groupe Hospitalier Paris Saint-Joseph, Le Plessis Robinson, France; e Medical Research and Innovation Unit, Inserm U999, Université Paris Saclay, Paris, France

**Keywords:** 3D, Aneurysm, Endovascular Bentall, Endovascular surgery, Simulation

## Abstract

Three-dimensionally (3D) printed models have been increasingly used in medicine. Few reports have focused on prototype experiments, especially in aortic surgery. Although endovascular repairs are routinely performed for thoracoabdominal aortic aneurysms and lesions involving the aortic arch, endovascular treatment of the ascending aorta is still at an early stage of development. Using a 3D model, based on patient computed tomography scans and manufactured by Biomodex (Paris, France), we performed a patient-specific rehearsal of an endovascular Bentall repair to treat an ascending aorta aneurysm involving the aortic root. We achieved a patient-specific proof of concept of a new technique using an in vitro 3D model.

Gaia et al^1^ recently described the first-in-human Bentall procedure performed through an endovascular approach. However, no dedicated endografts have yet been developed and validated for exclusion of aortic root aneurysms. We have designed an aortic endograft with coronary artery branches in collaboration with the Cook Medical planning center (Brisbane, Australia) to repair a complex aortic root in a patient with contraindications for conventional open surgical repair. We planned to exclude the aortic disease by implanting the custom endograft in combination with a transcatheter aortic valve replacement (TAVR) procedure.

Before performing this innovative and personalized procedure, a patient-specific three-dimensionally (3D) printed model (Biomodex, Paris, France) was developed to simulate the procedure in a real-life setup and provide a proof of concept of our therapeutic strategy.

## Methods

### Patient characteristics

A 69-year-old woman was referred to our institution for an increasing aneurysm in the ascending aorta involving the aortic root. Her medical history included an aortic valve repair (Perimount Magna Ease, 21 mm; Edwards, Lifesciences Corp, Irvine, Calif) combined with wrapping of the ascending aorta, performed 12 years earlier. The wrapping had failed and migrated distally. The patient was considered at high risk for redo sternotomy owing to her impaired left ventricular function, severe chronic obstructive pulmonary disease, and poor mobility related to left hemiplegia. The institutional review board reviewed the clinical protocol as a compassionate treatment and approved the preclinical simulation. The patient provided written informed consent for the report of her case.

### 3D printed model

**Fig 1:**
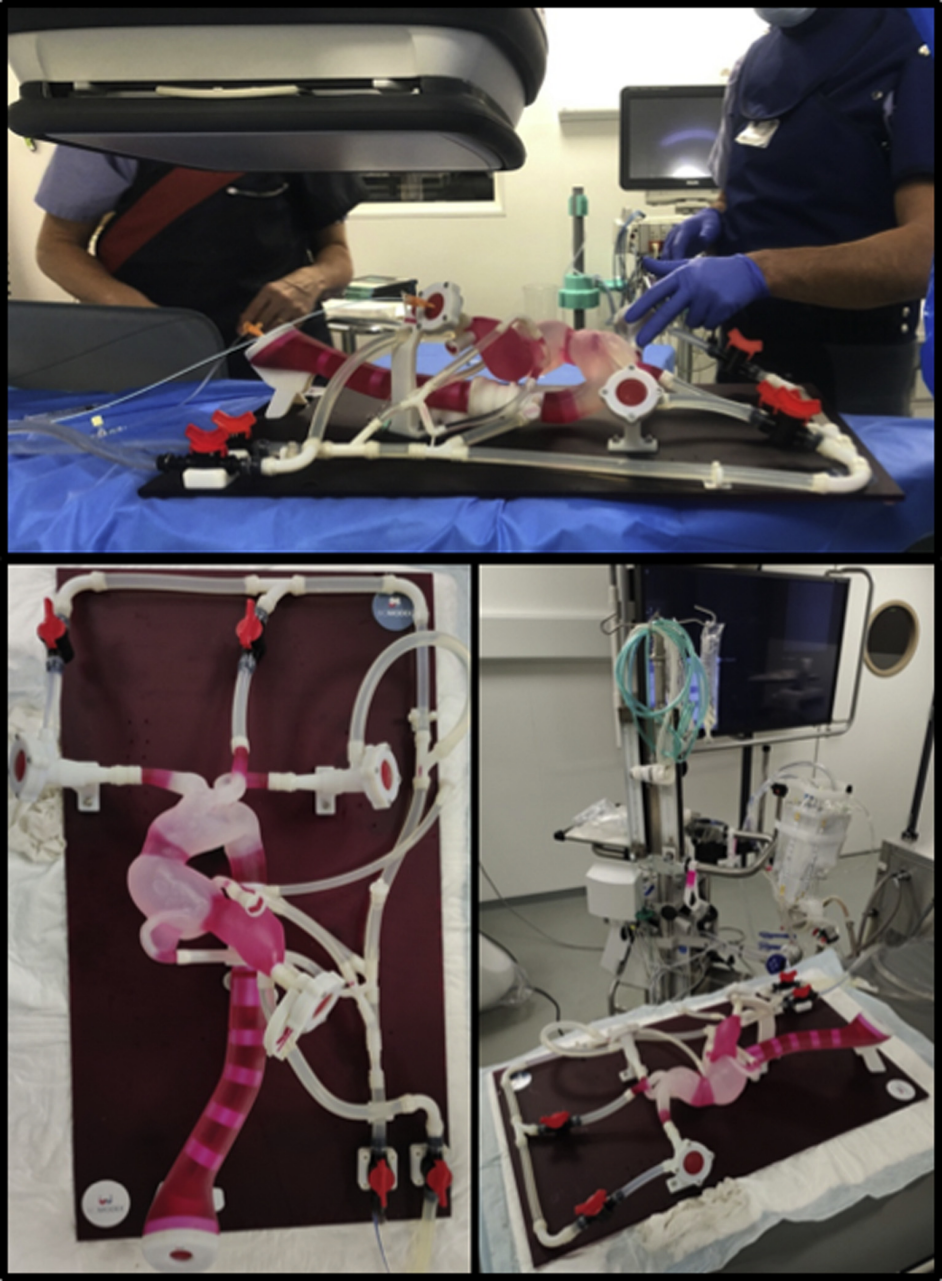
**A,** Three-dimensionally (3D) printed model shown under fluoroscopy flat panel. **B,** 3D printing model. **C,** 3D printed model connected to the centrifugal pulsatile pump.

The 3D printed model (Fig 1) was manufactured by Biomodex (Paris, France) using a Stratasys polyjet 3D printer (Rehovot, Israel). The training model was manufactured using information from the latest computed tomography (CT) scan of the patient. The imaging dataset was segmented using the CE-marked/Food and Drug Administration–approved Simpleware ScanIP software tool (Synopsys Inc, Mountview, Calif). The printed model replicated the patient’s anatomy three dimensionally from the femoral artery to the aortic valve bioprosthesis. The model was printed using a proprietary Biomodex algorithm called INVIVOTECH. This algorithm combines soft and rigid materials at the microscopic level, providing realistic biomechanical behavior of the 3D printed model.

### Hybrid room

The simulation procedure was performed at Marie Lannelongue Hospital (Paris Saclay University, Le Plessis Robinson, France) in a state-of-the-art hybrid room dedicated to preclinical experiments (Discovery IGS 730; GE Healthcare, Milwaukee, Wis). Advanced imaging applications, including image fusion guidance and cone beam CT were used.

### Hemodynamic features

Blood viscosity was mimicked by the proprietary BloodSim formula (Biomodex). The 3D model was connected to a centrifugal pump (Medos Deltastream DP3 system; Medos, Stolberg, Germany) to deliver anterograde pulse flow.

## Results

The procedure was performed as follows and is shown in the Supplementary Video.

**Fig 2:**
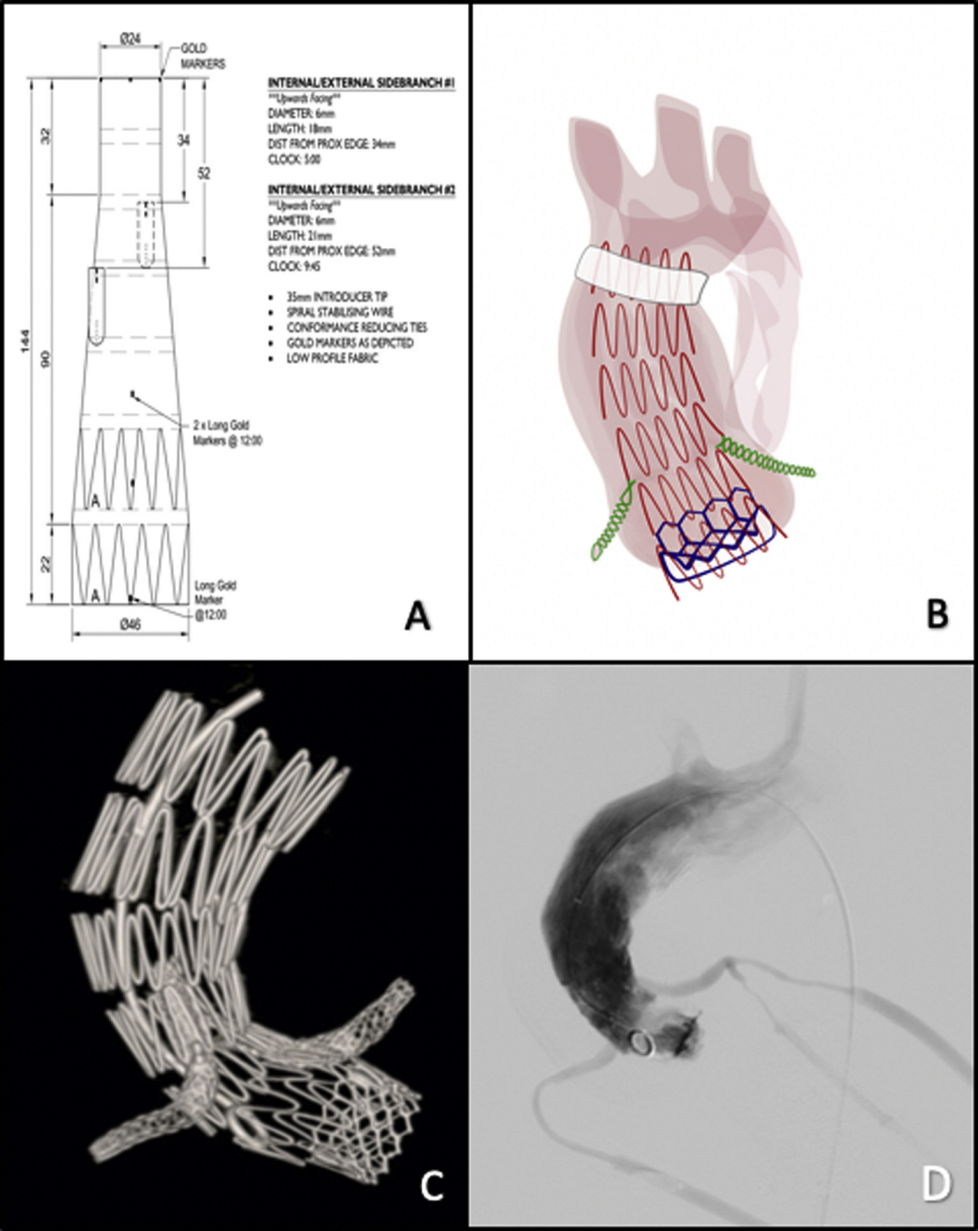
**A,** Schematic of the ascending branched endograft. **B,** Schematic of the final configuration of the different components. **C,** Completion non–contrast-enhanced cone beam computed tomography (CT) scan. **D,** Final angiogram showing aneurysm exclusion, patent coronary arteries, and the absence of paravalvular leaks.

1. Vascular access and angiography
  • A pigtail catheter was placed in the aortic root though a 5F sheath inserted from the right radial access.
  • A 16F introducer sheath (Dryseal; WL Gore & Associates, Flagstaff, Ariz) was placed into the arch lumen over a stiff wire from a left axillary access. This sheath was used during the procedure to perform the TAVR.
  • A 5F sheath was introduced through the apex of the left ventricle (LV).
  • An angiogram was performed to check fusion mask accuracy.
2. Placement of branched thoracic arch graft (Fig 2, *A* and *B*)
  • A 4-meter long wire was advanced through the 5F ventricular access sheath, through the aortic valve, and then snared from the femoral access site to create a through-and-through access wire.
  • Before inserting the delivery system, the markers of the endograft were checked under fluoroscopic guidance to adjust orientation and positioning.
  • The 24F delivery system was introduced over the guide wire until the tapered tip had passed through the aortic valve and into the LV.
  • An angiogram was performed to confirm appropriate positioning of the coronary branches according to their respective target vessels and to visualize the origin of the brachiocephalic trunk.
3. Graft deployment
  • While reducing cardiac (pump) output by 50%, the sheath was withdrawn until the graft was completely deployed.
  • The spiral stabilizing wire was then removed, along with the inner curve proximal attachments and proximal conformance ties.
  • The tip of the delivery system was retracted into the descending thoracic aorta.
4. TAVR
  • This step was conducted via the previously placed 16F left axillary sheath, before catheterization and stenting of the target vessels (coronary arteries). A wire was advanced directly from the left axillary artery sheath through the endograft and into the LV. A valve-in-valve TAVR procedure (20 mm, Sapien 3; Edwards Lifesciences, Irvine, Calif) was then performed over the wire. Axillary access was used to minimize the major aortic insufficiency period after endograft deployment through the aortic valve.
  • The seal was obtained by the endograft protruding within the LV through the aortic valve. It was then secured with a TAVR.
5. Cannulation of coronary arteries
  • A 6F, 90-cm introducer sheath was advanced into the endograft lumen from the femoral access.
  • A seeking catheter and guide wire were advanced to cannulate the first branch and its target vessel (left coronary artery).
  • The access wire was removed and replaced with a support wire (Rosen; Cook Medical, Bloomington, Ind).
  • The coronary arteries were cannulated using a 6F sheath, and the bridging stent was placed at its intended delivery site (protruding >5 mm within the coronary arteries).
  • Full overlap between the bridging stent and endograft branch was achieved.
  • The bridging cobalt chrome balloon expandable polytetrafluoroethylene covered stents (5 × 57 mm distally and 7 × 37 mm proximally; Begraft; Bentley, Hechingen, Germany) were molded.
  • The same maneuvers were then performed for the right coronary artery using the same type of bridging stents)
6. Final angiogram and cone bean CT
  • The final imaging studies showed no evidence of postprocedural endoleaks, neither on the distal landing zone nor at the level of TAVR. Patency of the coronary arteries and supra-aortic trunks was remarkable.
  • Technical success was confirmed on the completion angiogram and cone beam CT (Fig 2, *C*).

**Fig 3:**
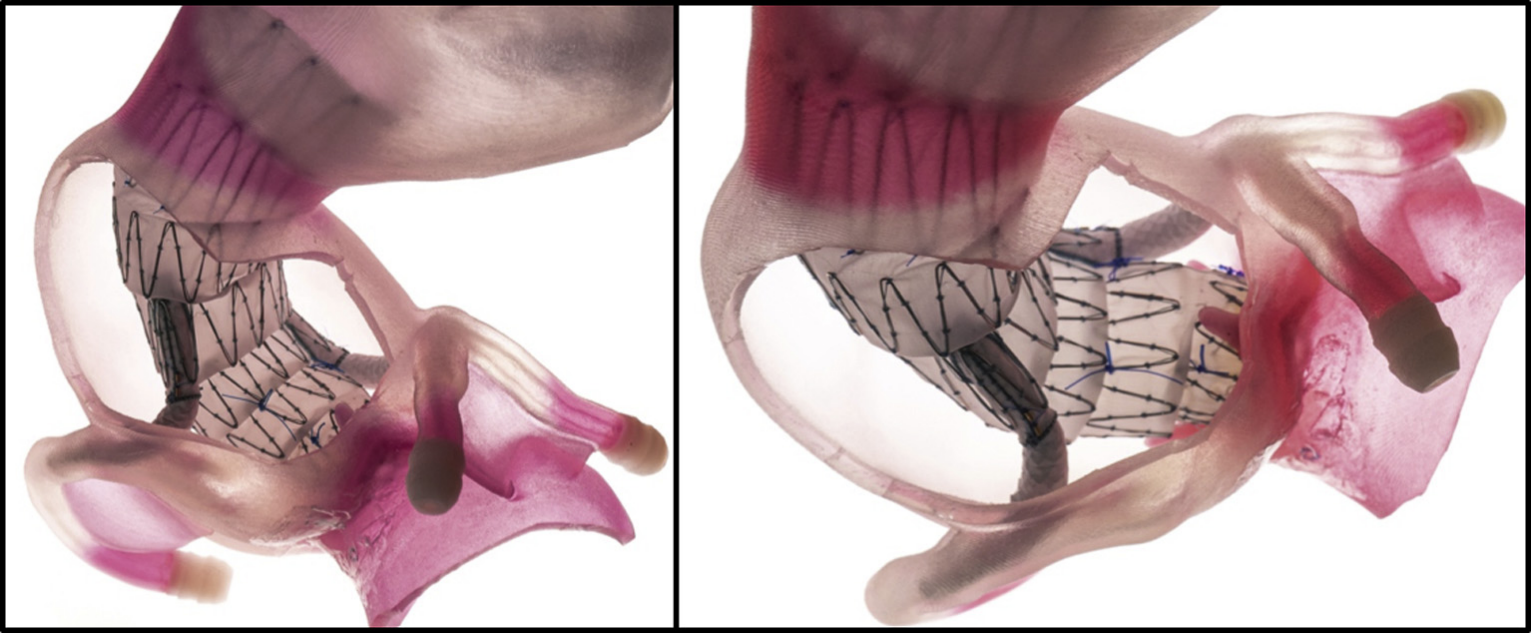
Three-dimensionally (3D) printed patient-specific model of ascending aorta aneurysm with endovascular Bentall graft after model autopsy.

The overall procedure was completed in 120 minutes. The TAVR procedure was completed 3 minutes after branched endograft deployment. We performed an “autopsy” of our endovascular Bentall repair (Fig 3) on the 3D printed model to confirm the appropriate deployment of the branched endograft, consistent with complete exclusion of the aortic root aneurysm (Fig 2, *D*).

## Discussion

This rehearsal session highlighted several disadvantages of this novel procedure such as the instability of the endograft during deployment, the challenging control of the branch’s orientation, and the need for support into the right coronary artery to advance the bridging stent.

We increased the endograft length by one extra stent after the rehearsal session because we had observed that once deployed in the ascending aorta curvature, the distal sealing zone was marginal to provide a long-term seal. We had also observed that right coronary catheterization was challenging and decided to overcome it with the use of a steerable sheath for that target vessel. During the “real” procedure, access to that right coronary artery had been challenging but eventually achieved. A right coronary spasm was observed after bridging stent inflation, followed by cardiac arrest that was rapidly rescued. Technical success (aneurysm exclusion and branch patency) was observed on the final angiogram. However, the patient died on postoperative day 2 of multiorgan failure.

The 3D printed model allowed us to perform a patient-specific proof of concept, the performance of which had been recommended to allow us to obtain approval from our ethics committee for the actual patient procedure. We strongly advocate for such patient-specific rehearsals before performing innovative clinical procedures, from both an ethical and a technical standpoint.

The Biomodex model with INVIVOTECH is a very life-like, realistic model, not only both visually and to the touch, but also in terms of haptic sensation during navigation. During the procedure, the clinical feedback was excellent and the confidence we realized through the model allowed us to adapt our planned method for the subsequent patient procedure. The usual manufacturing delay of these models is 15 days, which can be reduced to 5 days for urgent cases. The estimated cost is $6000. We believe that realistic approved 3D printed models are a valuable and reproducible alternative to in vivo models in the field of original custom device development and testing.

## Conclusions

We successfully achieved a patient-specific proof of concept by performing an endovascular treatment for aortic root aneurysm using a 3D printed patient-specific model consistent with an endovascular Bentall repair. Patient-specific rehearsal will play a major role in the ethical field of experimental procedures involving humans by its significant increase in patient safety.

## Appendix

Supplementary Video Endovascular Bentall repair procedure. *ICA,* Internal carotid artery.
